# SPectroscOpic prediction of bRain Tumours (SPORT): study protocol of a prospective imaging trial

**DOI:** 10.1186/s12880-020-00522-y

**Published:** 2020-11-23

**Authors:** Pamela Franco, Urs Würtemberger, Karam Dacca, Irene Hübschle, Jürgen Beck, Oliver Schnell, Irina Mader, Harald Binder, Horst Urbach, Dieter Henrik Heiland

**Affiliations:** 1 Department of Neurosurgery, Medical Centre, University of Freiburg, Breisacher Str. 64, 79106 Freiburg im Breisgau, Germany; 2 Department of Neuroradiology, Medical Centre, University of Freiburg, Breisacher Str. 64, 79106 Freiburg im Breisgau, Germany; 3 Specialist Centre for Radiology, Schoen Clinic, Vogtareuth, Germany; 4 Faculty of Medicine, University of Freiburg, Breisacher Str. 153, 79110 Freiburg im Breisgau, Germany; 5 Institute of Medical Biometry and Statistics, Faculty of Medicine and Medical Centre, University of Freiburg, Stefan-Meier-Str. 26, 79104 Freiburg im Breisgau, Germany

**Keywords:** Radiogenomics, Magnetic resonance spectroscopy, MRS, MR spectroscopy, Chemical chift imaging, 1H-MRS, MRI, Neuroradiology, Neurosurgery

## Abstract

**Background:**

The revised 2016 WHO-Classification of CNS-tumours now integrates molecular information of glial brain tumours for accurate diagnosis as well as for the development of targeted therapies. In this prospective study, our aim is to investigate the predictive value of MR-spectroscopy in order to establish a solid preoperative molecular stratification algorithm of these tumours. We will process a 1H MR-spectroscopy sequence within a radiomics analytics pipeline.

**Methods:**

Patients treated at our institution with WHO-Grade II, III and IV gliomas will receive preoperative anatomical (T2- and T1-weighted imaging with and without contrast enhancement) and proton MR spectroscopy (MRS) by using chemical shift imaging (MRS) (5 × 5 × 15 mm^3^ voxel size). Tumour regions will be segmented and co-registered to corresponding spectroscopic voxels. Raw signals will be processed by a deep-learning approach for identifying patterns in metabolic data that provides information with respect to the histological diagnosis as well patient characteristics obtained and genomic data such as target sequencing and transcriptional data.

**Discussion:**

By imaging the metabolic profile of a glioma using a customized chemical shift 1H MR spectroscopy sequence and by processing the metabolic profiles with a machine learning tool we intend to non-invasively uncover the genetic signature of gliomas. This work-up will support surgical and oncological decisions to improve personalized tumour treatment.

***Trial registration*:**

This study was initially registered under another name and was later retrospectively registered under the current name at the German Clinical Trials Register (DRKS) under DRKS00019855.

## Background

Gliomas are a very heterogenic group of tumours arising from glial cells in the central nervous system that are yet to be completely understood. Recent research advances have led to the characterization of different tumour subgroups based on molecular properties and have shown the necessity to individualize patient treatment according to each tumour entity. As the revised classification of brain tumours by the World Health Organization of 2016 gave an essential role to the specific genetic alterations, not only for prognosis or probability of chemosensitivity, but also for the actual classification of tumours, determining the molecular alterations of these tumours in a reliable way is the most important task in neuro-oncology today [1]. However, not all of the necessary molecular diagnostic methods are available throughout the world, and a medical expert in neuropathology may also be scarce. Hence the growing interest in developing tools to aid in the diagnosis and classification of brain tumours in a non-invasive and more efficient way. MR spectroscopy has long been a neuroradiological tool used to determine the metabolic alterations in different central nervous system pathologies [2–4]. Nonetheless, advances in MR imaging as well as in nuclear medicine imaging have lifted MR spectroscopy in the routine diagnostic workup of patients suggested to have a brain disease to another level. In many institutions, MR spectroscopy is used for research purposes only, or is regarded as a somewhat outed method with little diagnostic significance in addition to MRI [5–7]. Nonetheless, MR spectroscopy can deliver information beyond the metabolic profile of a lesion [5, 8].

Since 1990 several studies have been conducted to show the role of MR spectroscopy in the differentiation between various central nervous system lesions. In gliomas, these studies have been mostly based on the information provided by alterations in the metabolite ratios of i.e. Cho/Cr, Cho/NAA, and NAA/Cr [9–13]. However, the necessity of uncovering more information about the genetic profile of gliomas in a preoperative setting is the goal of research conducted in the field of MR spectroscopy [14, 15]

Following the discovery of IDH-mutations in humans, Choi et al. published a study in 2012 regarding the detection of 2-hydroxyglutarate specifically through MR spectroscopy in patients with gliomas, which accurately correlated with the presence of an IDH-mutation and the diagnosis of WHO-Grade II and III gliomas in adults [14]. This advance indicated that there is much more information hiding behind chemical shift imaging [15]. Nevertheless, MRS still has disadvantages which complicate its implementation into the routine workup of tumour patients. Some of these are the lack of experience in interpreting results, the need of a homogeneous magnetic field in order to discern the resonance frequencies of each specific metabolite, the low concentration of metabolites in tumour lesions in respect to water (e.g. by edema), making a weak signal given by the resonant frequencies, the requirement of the lesion not being in the proximity of bone structures (i.e. skull base) and the fact that more interest has been put into developing molecular diagnostics processes of tumour tissue itself [15–17].

Previous work done by our authors has shown that gliomas can be classified through MR spectroscopy characteristics [18], and has shown furthermore a feasible way in which a genetic profiling based on MR spectroscopy can be done with metabolic alterations identified by MR spectroscopy being correlated and classified according to certain aberrations in oncogenic pathways [19–21].

## Objectives

In the present study, the main objective of this research study is to correlate spectroscopic and genetic signatures of gliomas, and to apply a machine learning algorithm through which an integrative automated and accurate diagnostic work-up of these tumours can be developed.

## Methods/design

### Study setting

The present study will be conducted at the Departments of Neurosurgery and Neuroradiology of the Medical Centre – University Freiburg. Our hospital is a tertiary care centre with referrals of a wide region that encompasses the borders of southwest Germany, northwest Switzerland and northeast France.

### Trial design

The study is a 5-year, monocentre, single arm, prospective diagnostic study trial of patients undergoing diagnostics and treatment for WHO-Grade II, III and IV gliomas.

### Trial status

The first patient was recruited on the 1st of August of 2016. The expected date of completion is May 2021.

### Participants characteristics

#### Eligibility criteria

Patients undergoing diagnostics and treatment for WHO-Grade II, III and IV gliomas who match the following criteria will be included in the study: (1) age older than 18 years, (2) suspected glial tumour and (3) location of the lesion not near the skull base.

MRI and MRS will be performed by a team of radiology technician and neuroradiologist specially trained for the planning and analysis of MRI and MRS in brain tumours in the Department of Neuroradiology. Glioma surgery will be performed by the surgeons at the Department of Neurosurgery.

### Interventions

#### Intervention description

Following written consent, patients with a suspected glioma will receive preoperative anatomical (T1-weighted sequences with and without contrast enhancement, T2- and T2-FLAIR-weighted sequences) and chemical shift imaging (CSI).

After imaging acquisition, patients will undergo surgical glioma resection. Intraoperative tissue samples of the contrast enhancing tumour part will be obtained for histopathological and genetic analysis.

**Fig. 1:**
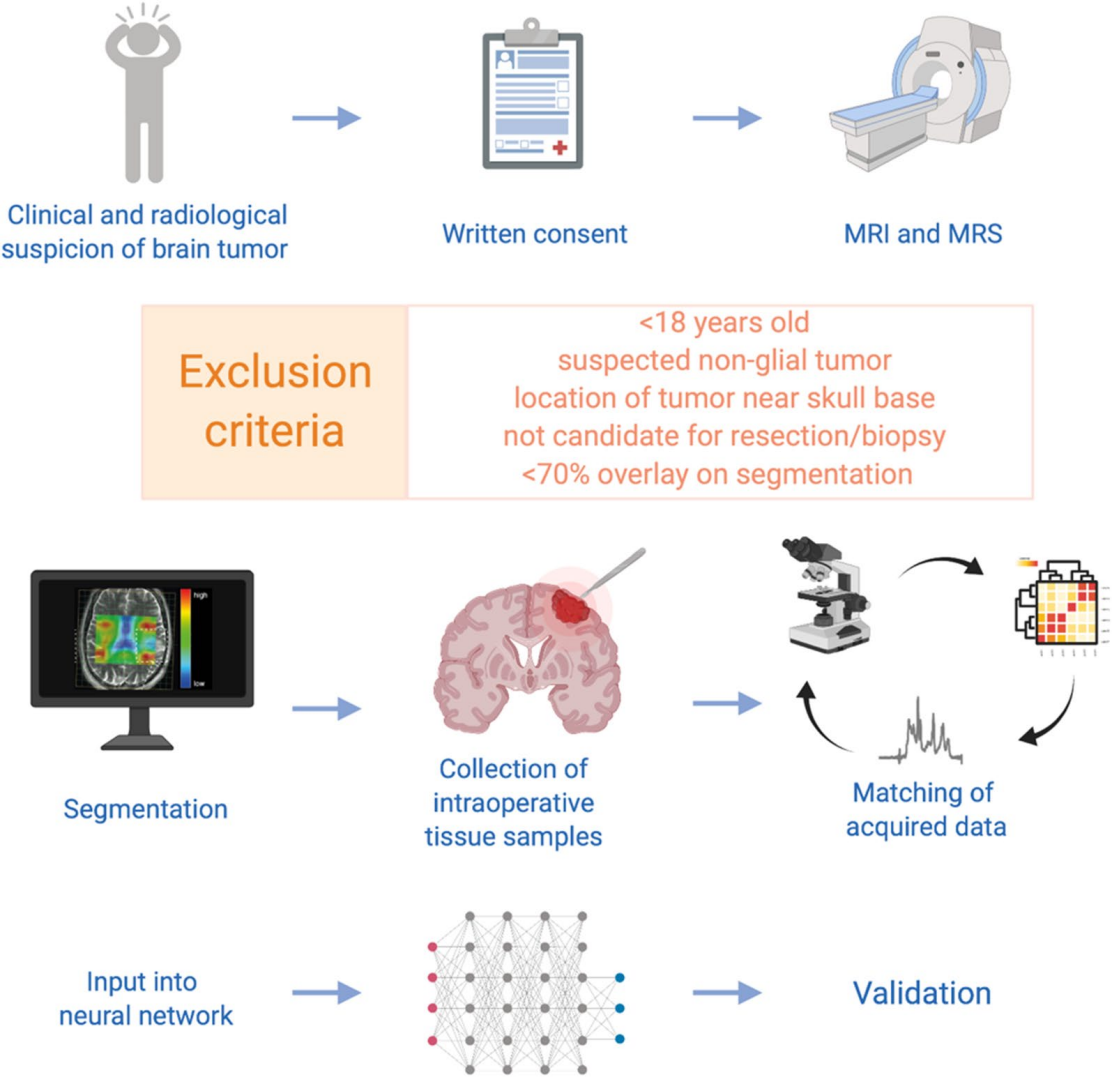
Concept of study design

Afterwards, spectral data will be matched to the final histopathologic diagnosis including metabolic profile as well as transcriptome analysis. The combined data of two thirds of the cohort will be used for training a machine learning approach, specifically an artificial neuronal network. The data of the remaining one third of the cohort will be consecutively used for validation of the algorithm (Fig. 1).

#### Imaging acquisition

Patients will be undergoing preoperative anatomical and *in-vivo* chemical shift imaging at a 3 T MR Magnetom Prisma scanner (Siemens, Erlangen, Germany) in the Department of Neuroradiology, Medical Centre—University Freiburg (details in Fig. 1.) Chemical shift imaging will be performed using the manufacturer’s provided chemical shift imaging sLASER sequence with a TE = 40 ms (Table 1). Partitions with a volume of interest (VOI) of 5 × 5x15 mm^3^ are intended to allow a more precise assignment of the area for intraoperative tissue sampling planning.

**Table 1:** Measurements of MR-spectroscopy

Mode	Duration (in minutes)
Preparation of the patient	05:00
T2-FLAIR 3D	07:52
T2-high resolution TSE	02:38
CSI-slaser, TE 40 ms (VOI: 5 × 5 × 15 mm^3^)	20:12
T1-MPRage post-contrast	03:54
Total	38:56

A timely evaluation will be performed using Syngovia® software (Siemens, Erlangen, Germany) and metabolic maps of twelve metabolites will be calculated as ratio to the contralateral normal appearing matter (NAM) and overlaid on high resolution T2- weighted images (0.63 × 0.63 × 2 mm^3^). An additional analysis by LC-Model will be performed and compared to Syngovia® software, in order to analyse the reliability of Syngovia® software. This step will ensure that the manufacturer’s software is able to analyse the spectroscopic data without the necessity for special spectroscopic software. Neuroradiologists with more than 20 years’ experience oversaw the imaging acquisition.

Individual spectra will be baseline corrected and fitted for twelve metabolites. Spectral data will then be aligned to the anatomical imaging and manually segmented into the following regions: normal appearing matter (NAM), ventricles (V) and lesion (L). Voxels with less than 70% overlay to a specific region will be excluded from further analysis.

### Tissue sampling

Navigation-based tissue sampling (Cranial Map Neuronavigation Cart 2, Stryker®, Freiburg, Germany) will be performed after previous planning by the surgical team at the Department of Neurosurgery of the Medical Centre—University Freiburg. Sampling locations will be aligned to the MR spectroscopy voxels by integration into the neuronavigation system. In high-grade glioma, sampling will be taken from the contrast-enhancing regions and in low-grade lesions from the T2-hyperintense regions. Tissue samples will immediately be snap-frozen in liquid nitrogen and processed. RNA will be prepared using the RNeasy Microarray Tissue kit (Qiagen®). An amount of 1.5 µg of RNA will be obtained for expression analysis. Neuropathological evaluation will be performed in the Department of Neuropathology, Medical Centre—University Freiburg according to their standards, including genome analysis (1p19q co-deletion and exome sequencing of IDH1/2).

### Transcriptome analysis (RNA sequencing)

RNA integrity will be measured using the Agilent RNA Nano Assay Agilent Bioanalyser 2100 (http://www.home.agilent.com) according to the manufacturer’s instructions. Sequencing libraries will be prepared according to the manufacturer’s instructions and sequenced on an Illumina HiSeq4000 sequencer. The resulting 150 bp paired-end reads will be quality-checked with FastQC. Sequencing data will be mapped to the human genome (hg38, UCSC, https://genome.ucsc.edu) with STAR (https://github.com/alexdobin/STAR). Followed by the alignment, transcripts with less than 10 reads will be removed. The given RNA counts will be normalized by DESeq2 based on a negative binomial distribution model [22]. STAR-Fusion will further process the output generated by the STAR aligner (above described) to map junction reads and spanning reads to a reference annotation set (https://github.com/STAR-Fusion/).

### Participant timeline

**Fig. 2:**
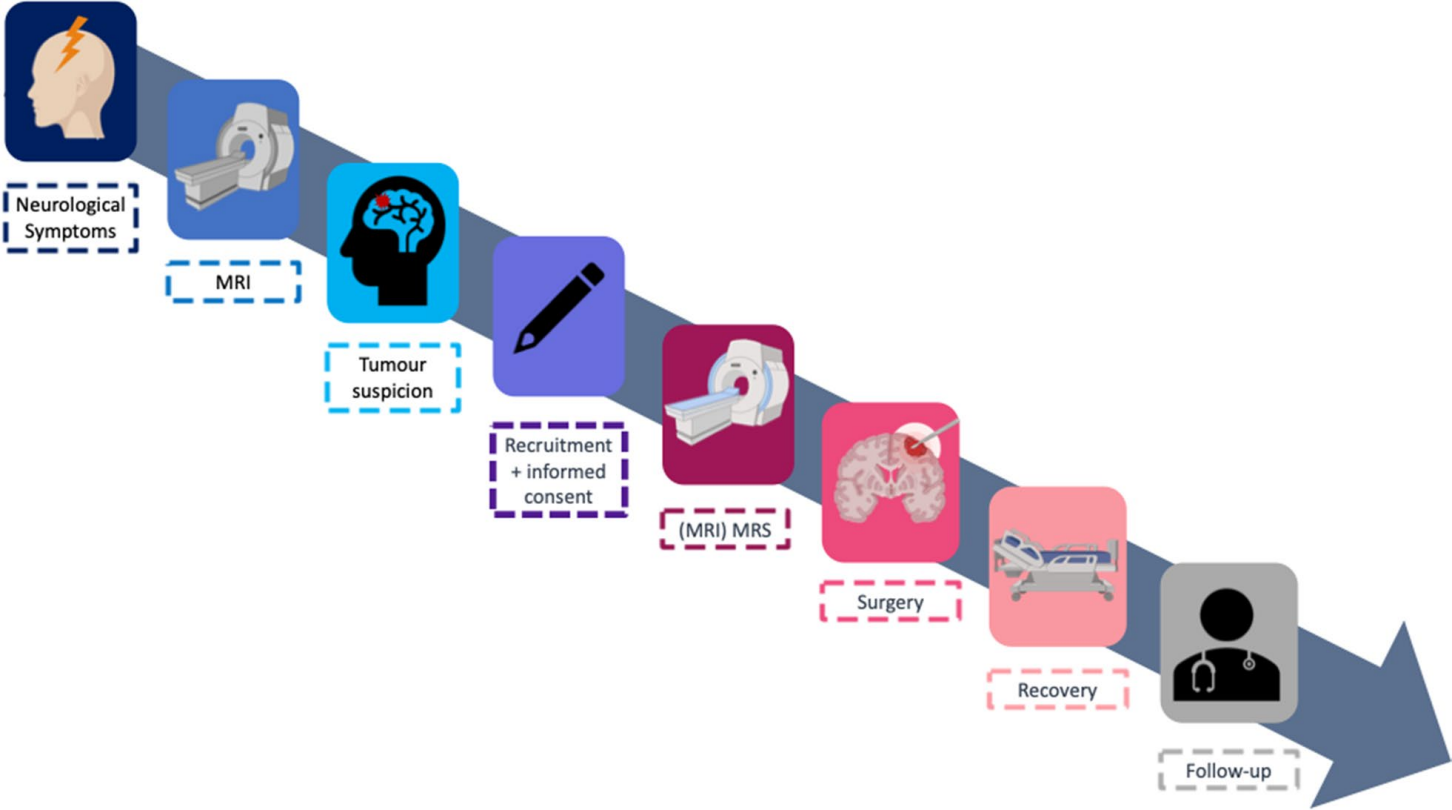
Participant timeline

A participant timeline is shown in Fig. 2.

Patient follow-up will be conducted according to our institution’s guidelines.

### Sample size

The number of prospective cases to be analysed is n = 300. The prospective case number is based on the counselling by Dr. Harald Binder of the Institute of Medical Biometry and Statistics, Faculty of Medicine and Medical Centre—University of Freiburg.

The imaging dataset will be trained by genetic profiling and result in a new “radiogenomic classification” (n = 200 in training set). This new classifier will be compared to the histopathological “gold standard” and tested in an independent validation set (n = 100). Additional survival analysis will be performed by Cox Proportional-Hazards Model. We expect to enrol 300 patients in the study.

### Data collection and management

#### Data management

All data material will be recorded with our institution’s own patient ID and will be unidentifiable outside the institution. The data will be stored electronically within our institution’s server and deleted 10 years after the project’s end.

### Confidentiality

Only the primary investigators will have access to the patients’ entire information (names, birthdays and so forth). Data will be protected according to German and European laws. Data quality will be checked by two independent researchers of the neurosurgical team.

### Plans for collection, laboratory evaluation, and storage of biological specimens for genetic or molecular analysis in this trial or future use

All patients’ tumour samples (used for histopathological, genetic and molecular analysis) will be stored at the Tumour Bank of the Department of Neurosurgery, Medical Centre – University Freiburg according to our institution’s guidelines and adhering to German and European Laws.

### Statistical methods

#### Biostatistics

The sample size has been previously determined by the Institute of Medical Biometry and Statistics based on prior experience [23]. It is of note, that there are to date no formal approaches for sample size calculation for complex multi-marker setting, as considered here.

The transcriptome data will be used to predict the expression subgroup of each patient. Data analysis will be performed by the Microenvironment and Immunology Research Laboratory at the Medical-Centre University of Freiburg. Obtained transcriptomic profiles will be analysed by the *RawToViz* pipeline (www.github.com/heilandd), and results will be available for the Vis_Labv.1.5 software (https://heilandd.shinyapps.io/Vis_Lab1/). To predict the subgroup affiliation, we use the state analysis based on a model first presented by Neftel et al in 2019 [24].

### Artificial neural network algorithm

For training the neural network algorithm, 120 spectroscopic samples with 256 voxels (16 × 16 matrix) providing twelve metabolites each (input layer array of 120 × 256x12), resulting in 368,640 subsets, will be used. A deep learning approach will be used for uncovering metabolic patterns and linking these to a categorical output. In the first unsupervised step, spectroscopic data of the first half of the 120 samples will be analysed according to their correlation structure. Specifically, we will use partitioned deep Boltzmann machines (DBMs) [23], which are particularly suited for applications with small sample sizes, to learn the distribution of metabolite measurements in samples of different classes. This will result in one DBM per class, where each DBM provides a compression of the information into a small number of top-level hidden network nodes. Subsequently, measurements from a new patient could be fed into each DBM, resulting in several compressed representations. We intend to use an additional DBM for linking the joint output of the per-class DBMs to the class labels.

### Interim analyses

An interim analysis is planned after n = 120 patients (30,720 voxels). The data of this cohort will then be used for the training of the neuronal network algorithm.

### Plans to give access to the full protocol, participant level-data and statistical code

The research team intends to make the complete genomic and imaging datasets available for the whole research community by providing it on a suitable portal (i.e. The Cancer Genome Atlas (TCGA) as well as The Cancer Imaging Archive (TCIA)).

## Discussion

Our study aims to develop a machine learning tool that can bring forth and correlate the metabolic signature of a glioma to its genetic profile. The metabolic signature will be derived from a readily available customized 3 T 1H chemical shift MR spectroscopy sequence. By using an open-software tool and a customized MRI sequence we intend to standardize the diagnostic work-up of gliomas and make it applicable even in places where genetic testing and therefore thorough diagnostic might be difficult to implement.

## Data Availability

The datasets and algorithms resulting and utilized in this study can be available upon request to the corresponding author.

## Abbreviations

MRS: MR-spectroscopy: magnetic resonance spectroscopy, chemical shift imaging
WHO: World Health Organisation
Cho: Choline
Cr: Creatine
NAA: N-acetyl-aspartate
IDH: Isocitrate dehydrogenase
CNN: Convolutional neural network
NAM: Normal appearing matter
VOI: Volume of interest
IDH: Isocitrate dehydrogenase
TE: Echo time
GS: Gene significance
MM: Module membership
kME: Intramodule connectivity
DBM: Deep Boltzman machine
